# Genotypic diversity of merozoite surface antigen 1 of *Babesia bovis* within an endemic population

**DOI:** 10.1016/j.molbiopara.2010.03.017

**Published:** 2010-08

**Authors:** Audrey O.T. Lau, Karla Cereceres, Guy H. Palmer, Debbie L. Fretwell, Monica J. Pedroni, Juan Mosqueda, Terry F. McElwain

**Affiliations:** a Programs in Genomics and Vector-borne Diseases, Department of Veterinary Microbiology and Pathology and School for Global Animal Health, Washington State University, Pullman, WA 99164-7040, USA; b National Center for Disciplinary Research in Veterinary Parasitology (CENID-PAVET)-INIFAP, Jiutepec, Morelos, Mexico

**Keywords:** MSA, merozoite surface antigen, RAP, rhoptry-associated protein, VMSA, variable merozoite surface antigen, HVR, hypervariable region, VESA, variant erythrocyte surface antigen, *Babesia bovis*, Merozoite surface antigen-1, Population genetics

## Abstract

Multiple genetically distinct strains of a pathogen circulate and compete for dominance within populations of animal reservoir hosts. Understanding the basis for genotypic strain structure is critical for predicting how pathogens respond to selective pressures and how shifts in pathogen population structure can lead to disease outbreaks. Evidence from related Apicomplexans such as *Plasmodium*, *Toxoplasma*, *Cryptosporidium* and *Theileria* suggests that various patterns of population dynamics exist, including but not limited to clonal, oligoclonal, panmictic and epidemic genotypic strain structures. In *Babesia bovis*, genetic diversity of variable merozoite surface antigen (VMSA) genes has been associated with disease outbreaks, including in previously vaccinated animals. However, the extent of VMSA diversity within a defined population in an endemic area has not been examined. We analyzed genotypic diversity and temporal change of MSA-1, a member of the VMSA family, in individual infected animals within a reservoir host population. Twenty-eight distinct MSA-1 genotypes were identified within the herd. All genotypically distinct MSA-1 sequences clustered into three groups based on sequence similarity. Two thirds of the animals tested changed their dominant MSA-1 genotypes during a 6-month period. Five animals within the population contained multiple genotypes. Interestingly, the predominant genotypes within those five animals also changed over the 6-month sampling period, suggesting ongoing transmission or emergence of variant MSA-1 genotypes within the herd. This study demonstrated an unexpected level of diversity for a single copy gene in a haploid genome, and illustrates the dynamic genotype structure of *B. bovis* within an individual animal in an endemic region. Co-infection with multiple diverse MSA-1 genotypes provides a basis for more extensive genotypic shifts that characterizes outbreak strains.

## Graphical abstract

Investigation of the population dynamics of pathogens contributes to a better understanding of disease transmission. Herein, the extent of *Babesia bovis* genotypic strain diversity within a defined cohort is reported.

## 1 Introduction

The phylum Apicomplexa contains several highly significant vector-borne pathogens of humans and animals prevalent in the same areas, including *Babesia*, *Plasmodium* and *Theileria*, the causative agents of babesiosis, malaria and theileriosis, respectively. Diversity in surface protein genes is well described in all three parasites [12]. In contrast to the rapid generation of change in some surface proteins (e.g. *Plasmodium falciparum* and *Babesia bovis*) [2], other surface proteins are more stable and result in readily identifiable strains that can be genotypically and phenotypically defined [18,24].

*Babesia bovis* merozoite surface antigen (MSA)-1 has been used as a genetic marker to demonstrate the presence of variant *Babesia* strains in different geographic regions [14,19]. MSA-1 is a member of the variable merozoite surface antigen (VMSA) gene family [10], is expressed on the merozoite surface and contains immunodominant B and CD4^+^ T cell epitopes [6]. Of importance, the MSA-1 genotype in isolates from acute, clinically affected animals that have been previously immunized with a live, attenuated vaccine strain (vaccine breakthroughs) is always distinct from the vaccine strain, indicating that MSA-1 is a marker of diversity associated with vaccine breakthroughs [14]. Consistent with a role of evading immunity induced against a distinct vaccine strain, antibodies generated against MSA-1 in one strain may completely lack cross-reactivity with MSA-1 in a geographically distant strain [16].

Vaccine breakthroughs occur infrequently. Therefore, it is plausible to speculate that genotypic diversity represented by MSA-1 may be minimal within the reservoir animal population and over time. This hypothesis is supported by both *in vitro* and experimental *in vivo* passage studies in which MSA-1 sequence was stable (T. McElwain, unpublished observation). However, these studies do not accurately mimic the selective pressure for diversity within a reservoir population or the dynamics of transmission within an endemic region. Alternatively, MSA-1 diversity may be occurring within a reservoir population without clinical impact. Consequently, we determined the MSA-1 diversity within an endemically infected reservoir population and in individuals within the population over time. Herein, we report testing the hypothesis of minimal genotypic diversity and discuss the results in the context of how genetic diversity leads to disease outbreaks.

## 2 Materials and methods

### 2.1 Animal population and samples

Blood samples were collected twice at a 6-month interval from a single herd of animals that were predominantly adult at the Experiment Station El Verdineño/National Institute of Forestry, Agricultural and Animal Research (INIFAP), Nayarit, Mexico. Located subtropically, Nayarit, Mexico (lat: 23°05′ N and 20°36′ S; long: 103°43′ E and 105°46′ W) is situated on the central west coast, bordering the Pacific Ocean. The blood was collected into EDTA- or sodium heparin-containing Vacutainer^®^ tubes by jugular venipuncture. Serum collected from anticoagulant free blood was tested by indirect fluorescence assay for *B. bovis* antibodies. Whole blood from serologically positive individuals was aliquoted in 500 μl and used to extract DNA. A total of 49 serologically positive animals were analyzed from the initial sampling and a subset of 31 of these animals was analyzed at the second time point.

### 2.2 Genomic DNA extraction

All centrifugation steps were carried out at 4 °C. Whole blood was centrifuged at 0.8 × *g* for 20 min to concentrate cells. A 200 μl aliquot of concentrated cells was washed 2× with sterile 1× PBS and resuspended in 50 μl of fresh 1× PBS. Puregene RBC Lysis Solution (300 μl) was added to cells, incubated for 1 min at room temperature and centrifuged for 5 min at 16,000 × *g*. The pellet was resuspended in 500 μl Puregene Cell Lysis Buffer containing 1 mg proteinase K ml^−1^ and incubated overnight at 56 °C. Samples were placed on ice to cool for 2 h. Ice-cold protein precipitation solution (200 μl) was added and samples were vigorously vortexed and incubated on ice for 5 min, then centrifuged for 3 min at 16,000 × *g* to precipitate proteins. The supernatant was transferred to a new tube and precipitated with 100% isopropanol containing 40 μg glycogen ml^−1^. The nucleic acid pellet was washed 1× with 70% ethanol and resuspended in 25 μl of TE buffer, pH 8.

### 2.3 PCR amplification of *msa*-1 gene

**Fig. 1:**
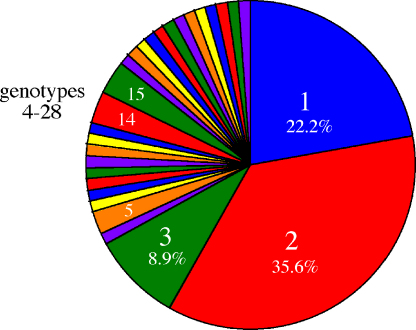
Frequency distribution of 28 distinct *B. bovis* MSA-1 genotypes isolated from two separate samplings of a *B. bovis* infected herd of cattle in Nayarit, Mexico. A distinct genotype is defined by greater than or equal to a single amino acid change from the rest of the genotypes.

Parasite DNA was amplified with oligonucleotide primers *msa*-1 forward (5′-ATGGCTACGTTTGCTCTTTTCATTTC-3′) and *msa*-1 reverse (5′-AAATGCAGAGAGAACGAAGTAGC-3′) designed from the Texas T2Bo isolate of *B. bovis* (XP_001608956.1). These two primers anneal to highly conserved 5′ and 3′ terminal sequences of *msa*-1 [13]. PCR conditions were 95 °C for 3 min, followed by 35 cycles of 95 °C for 30 s, 55 °C for 30 s, 72 °C for 90 s, and a final extension of 72 °C for 7 min. *B. bovis* Mo7 biological clone DNA and water were used as positive and negative amplification controls, respectively. Proof-reading Taq polymerase (Invitrogen) was used throughout all reactions. PCR was set up in triplicate and the resulting amplicons were cloned into pCR4-TOPO cloning vector (Invitrogen). Plasmid DNA from 30 clones from each sample was extracted using QIAprep^®^ Spin Miniprep Kit (Qiagen) and sequenced with M13 forward (5′-GTAAAACGACGGCCAG-3′) and M13 reverse (5′-CAGGAAACAGCTATGAC-3′) primers using standard ABI chemistries. All colonies extracted from each sample were sequenced to ensure that variant MSA-1 products, if present, could be detected. All preliminary sequence analyses performed in this study were conducted at the nucleotide and amino acid levels; however, determination of genotypes and subsequent distributions within the study cohort were conducted at the amino acid level only since many of the single nucleotide polymorphisms observed were synonymous. Overall distribution of genotypes was determined based on the initial sequences/sample (Fig. 1). The proportion of genotypes detected in each sample was not used in the analysis, only their presence. Additional sequencing of some samples was carried out to confirm that genotypes obtained during the first round of sequencing were reproducible and that no minor genotypes were detected the second time around. Power calculations were performed using the binomial theorem to estimate probability of detecting an individual parasite genotype. Thirty clones per sample provided a 95% probability of detecting a genotype present in the population at a level as low as 9.5%. Statistical significance of genotype changes within an animal between the two sampling periods was calculated using binomial test for equal proportions with *p* ≤ 0.05. To determine potential sequence variability of MSA-1 *in vitro*, 500 ng of gDNA from three separate batches of established long-term blood cultured *B. bovis* T2Bo strain was used to amplify *msa*-1. Thirty-three clones were selected and sequenced as described above. Analysis of sequence data of MSA-1, including phylogeny and ranges of amino acid identities within and between cluster groups were determined using MacVector^®^ with Assembler, Version 10.0. Multiple amino acid alignments were performed using ClustalW as the default setting on MacVector and Neighbor joining tree analysis was predicted using Bootstrap with random tie breaking as a feature. Poisson distribution that would measure the distances between nodes and gap sites was ignored.

### 2.4 PCR amplification of *rap*1.1 gene

Rhoptry-associated protein (RAP)1.1 gene was amplified in selected samples. These include randomly selected samples that contained a single MSA-1 genotype and all samples that contained multiple MSA-1 genotypes in single blood samples. *B. bovis* DNA extracted from the blood samples was amplified with the oligonucleotide primers *rap*1.1 forward (5′-ATGAAAAAAGCAGTATTTACTACGTTAATAGG-3′) and *rap*1.1 reverse (5′-TCATATATTGTTTATGTTTGATGCGAATTCAT-3′) designed from the *B. bovis* Texas T2Bo isolate (XP_001610909.1) under the same conditions as described for *msa*-1 PCR amplification. This primer set anneals to the 5′ and 3′ terminal coding sequence of *rap*1.1. All amplifications were performed in triplicate. The amplification products were cloned, sequenced and analyzed using the same methods as described above. Statistical significance of RAP1.1 protein sequence changes in the cohort was calculated using the Fisher exact test with *p* ≤ 0.01.

## 3 Results

### 3.1 Genetic variation of MSA-1 within an endemic population

**Fig. 2:**
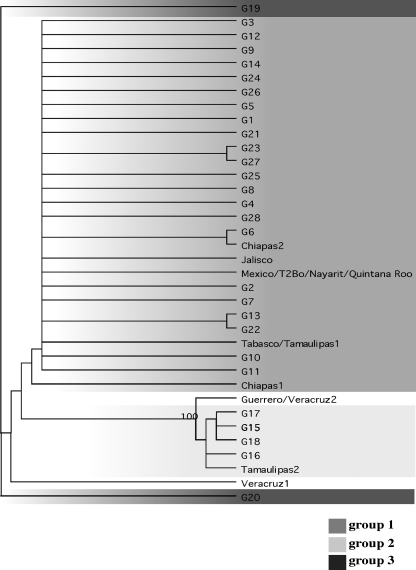
Neighboring-joining tree depicting the relationships between known *B. bovis* MSA-1 genotypes in Mexico and the 28 *B. bovis* MSA-1 genotypes isolated within an endemic cattle herd in this study. The phylogeny analysis was carried out with 1000 replicates. Tie of tree was randomly selected and not dependent on the order of the aligned sequences; treatment of gaps was distributed proportionally. Number shown at the node represents the percentage of sequence identity. Based on sequence homology, these MSA-1 genotypes as well as those newly identified genotypes can be grouped into three clusters. Group 1 consists of genotypes 1–14, 21–28 (dark grey) while groups 2 and 3 consist of genotypes 15–18 (light grey) and 19–20 (black), respectively.

In order to determine genotypic variability of *B. bovis* within a reservoir population over time, 49 individual infected animals within a single herd were analyzed for the presence of MSA-1, and 31 of these animals were tested a second time 6 months later. Analysis of MSA-1 sequences amplified from samples identified 28 distinct MSA-1 genotypes circulating within this animal population (Nayarit, Mexico) during a 6-month period (Fig. 1). Based on sequence homology, all 28 MSA-1 genotypes clustered into three main groups (Figs. 2 and S1). The ranges of amino acid identities within each cluster group were 96–99%, 98–99% and 89–91% for cluster groups 1, 2 and 3, respectively, while percentages of amino acid identities between cluster groups were 49%, 40% and 39% between cluster groups 1 and 2, 1 and 3, and 2 and 3, respectively. The major differences among genotypes in separate clusters occurred between amino acid 263–272, a region of MSA-1 which has previously been classified as hypervariable (HVR) [13]. Within each cluster group of genotypes, the number of amino acid substitutions ranged from one to four. These amino acid changes were distributed throughout the protein (Fig. S1).

In order to rule out that the observed sequence variation was a result of PCR artifact or sequencing error, the same samples were amplified with primers for *rap*1.1, a strictly conserved gene [22], and 30 amplicons per selected sample were sequenced. The results confirmed that RAP1.1 sequences were invariant among individual animals in contrast to the genotypic diversity of MSA-1 in these same animals. In addition, to determine whether sequencing or PCR artifacts might be gene specific, *msa*-1 was amplified and 33 clones from each of three separate long-term *in vitro* cultured *B. bovis* infected cell lines were sequenced. All sequences obtained from *in vitro* cultured *B. bovis* were identical. The absence of MSA-1 and RAP1.1 sequence variation obtained from cultured *B. bovis* DNA and field isolates, respectively, provides evidence that MSA-1 is stable *in vitro*, and that MSA-1 sequence variation identified within the infected reservoir population represents genotypic diversity in the population.

Within the 28 unique MSA-1 genotypes, genotype 2 predominated, representing 36% of the total sequences identified at both time points (Fig. 1). Genotypes 1 and 3 constitute the second and third most common genotypes, respectively (Fig. 1). They differ from genotype 2 by a single amino acid substitution at position 173. This position was one of the most common substitutions identified during the sampling period (16/28 genotypes contain this substitution). The second most common substitutional position was at position 334 (Fig. S1). With the exception of genotypes 1, 2, 3, 5, 14 and 15, many of the detected genotypes were rare in all the animals tested, with only one animal identified with each genotype. The genotype 2 sequence that dominated within this population is identical to previously identified strains in Quintana Roo (EF640946), an endemic region in the southeast region of the Yucatan peninsula, Nayarit (EF640953), and “Mexico” (EF640943). Furthermore, genotype 2 (as well as genotypes 1 and 3) is also almost identical (>99% identity) to MSA-1 identified in Chiapas, Jalisco, Tabasco and Tamaulipas 1 (Figs. 2 and S2). In contrast, genotypes 15–18 are similar to a MSA-1 sequence isolated in Tamaulipas (Tamaulipas 2) (Fig. 2). All genotypes isolated contained conserved motifs YFK and for a majority of these genotypes, GNLx motif, at positions 167–169 and 300–306, respectively (Fig. S1). Specifically, GNLx motif falls between positions 300–303 for genotypes 1–14 and 21–28 but for G15–18, this motif lies between positions 303–306. G20 is the only genotype with undetectable GNLx conserved motif. While the functions of these previously identified motifs have not been determined, they are conserved among many geographic isolates of *B. bovis* [14].

### 3.2 Temporal shift of MSA-1 genotypes within a population

**Fig. 3:**
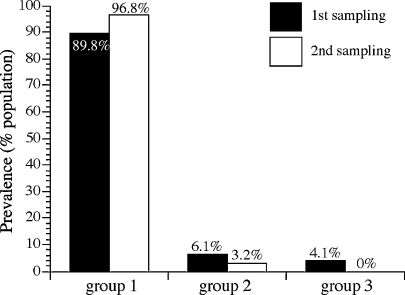
Distribution of *B. bovis* MSA-1 genotype groups in samplings 1 and 2. Prevalence of genotype groups in the first sampling is shown in black and that in second sampling is shown in white. Clusters of genotype based on sequence homology were grouped into three groups. Genotypes 1–14, 21–28 represent group 1 genotype while groups 2 and 3 include genotypes 15–18 and genotypes 19–20, respectively.

Fig. 3 shows the prevalence of genotypes analyzed by cluster groups (see Fig. 2 for cluster group definition). The group 1 cluster (genotypes 1–14 and 21–28) was dominant in both samplings, followed by groups 2 (genotypes 15–18) and 3 (genotypes 19–20). Genotypes 19 and 20 are grouped together as a cluster because they share some sequence similarities (e.g. insertion between positions 28–31) that are absent in the remaining 26 genotypes. Percent of amino acid identity between genotypes 19 and 20 is between 90% and 92% (Fig. S1). The prevalence of group 1 increased from 90% to 97% while group 2 was proportionally decreased and group 3 was absent in the second sampling. However, these changes did not reach a level of statistical significance due to the small number of samples containing each cluster group.

**Fig. 4:**
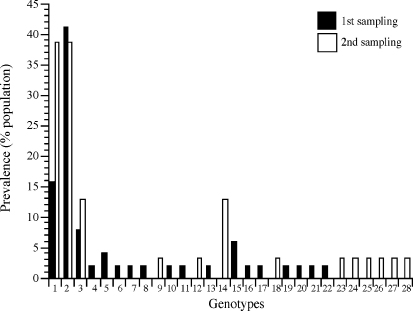
Distribution of 28 distinct *B. bovis* MSA-1 genotypes in both samplings of the population. Genotype prevalence in first sampling is shown in black while genotype prevalence in subsequent sampling is shown in white. Samples were collected 6 months apart.

Within cluster group 1, genotype 2 was the predominant MSA-1 at the first sampling time point (41%), while genotype 1 was most prevalent in the population at the second sampling 6 months later (Fig. 4). Genotype 2 prevalence decreased to 36% in the second sampling, while genotype 1 increased from 16% in the first sampling to 39% 6 months later. The prevalence of genotype 3 also increased from the first to second sampling. In other cluster groups, genotype 14 was prevalent in the second sampling, increasing by 13% within the infected population. This genotype was not detected in the first sampling (similar to genotypes 9, 12, 18, 23–28). In contrast, genotypes 4–8, 10–11, 13, 15–17 and 19–22, identified during the first sampling, were undetected in the subsequent sampling (Fig. 4). All genotypes identified in the first sampling contained tyrosine at aa173 and/or leucine at aa334. However, 6 months later, the majority of the resulting genotypes contained histidine at position 173 and phenylalanine at position 334.

### 3.3 Temporal genotypic shift of MSA-1 in individual animals

Among the 49 infected animals, 31 were examined over a 6-month period to determine if genotype shifts occur within an individual. Of these 31 animals, there was a genotypic change in 20 animals over the 6-month period. When a genotype was detected in the second sampling that was not present in the first sampling, re-sequencing of an additional 30 clones from the first sample was performed when adequate sample remained. In no animals was the newly appearing genotype from the second sample found in the first sample upon re-analysis. These genotypic shifts within animals over both sampling periods were significant (*p* = 0.048) using the binomial test of equal proportion, indicating that the observed changes may represent the appearance of new genotypes in individual animals or a different mixture of the same ones. The 11 animals that maintained the same genotype were infected with *B. bovis* MSA-1 genotype 2.

Within the 20 animals with genotype shifts, seven had inter-group changes with amino acid substitutions occurring primarily between positions 215 and 300 (Table 1). The remaining shifts were intra-group changes with relatively few substitutions (data not shown). Within the inter-group changes, six of seven (86%) were to group 1, consistent with the overall increased prevalence of group 1 genotypes found in the population at the second sampling (Table 1). All inter- and intra-group changes included the substitution, Y173H (data not shown). An additional frequent position for substitution was amino acid 334, where phenylalanine was found in 19 of 28 total MSA-1 genotypes while the remaining seven contain leucine at position 334.

Five individual animals contained multiple MSA-1 genotypes at a single time point. All genotypes found within an individual animal at the same time point were within the same cluster group. In four of these five animals, there was no overlap of genotypes over time and in two of these four samples, inter-group shifts occurred. All inter-group shifts were from cluster group 2 to 1.

## 4 Discussion

MSA-1 sequence diversity among geographically distinct *B. bovis* isolates has been well documented, and has been associated with vaccine breakthrough in areas where attenuated vaccines are routinely used. However, vaccine breakthroughs are infrequent, consistent with overall population stability punctuated by rare genetic change or introduction and establishment of a new strain. In addition, MSA-1 has been stable during multiple experimental passages of *B. bovis* strains through animals, including tick transmissions [5]. Consequently, we hypothesized that MSA-1 sequence diversity would be minimal within a geographically restricted reservoir host population and that the dominant genotype(s) would be stable over time. In this current study, we reject this hypothesis based on the identification of 28 genotypically distinct MSA-1 genotypes circulating in the study cohort, and clear shifts in genotypes over a 6-month period.

That the multiple *msa*-1 genotypes detected in this study represent true variants in the population and not artifacts generated by either amplification or sequencing errors is supported by two independent lines of evidence. First, amplification of a second target gene, *rap*1.1, from the same blood samples (all samples containing more than one MSA-1 sequence type and random samples containing a single variant MSA-1) revealed absolute identity. A Fisher exact test further confirmed the significance of this result (*p* < 0.0001), consistent with the conservation of RAP1.1 among *B. bovis* strains and indicates that the samples themselves were not prone to the generation of amplification or sequencing errors. Second, repeated amplification of MSA-1 from *in vitro* culture revealed absolute identity. This is consistent with the stability of MSA-1 *in vitro* and in experimental transmissions and indicates that neither MSA-1 nor the procedures used to amplify MSA-1 are inherently error prone. Nearly all (26/28) MSA-1 genotypes identified are very similar to MSA-1 genotypes previously detected throughout Mexico, with two genotypes, 6 and 16, identical to isolates from Chiapas (EF640945) and Tamaulipas (EF640949), respectively. Further, with the exception of the isolates designated Veracruz 2/Guerrero (EF640951/EF640947) and Tamaulipas 2 (EF640949), whose sequences are more divergent and belong to the cluster group 2, all genotypes previously identified in Mexico belong to cluster group 1 identified in this study. A recent study by Genis et al. using MSA2a and 2b (other members of the VMSA family) also demonstrated that *B. bovis* isolates in Mexico group into “clades” with no geographical association of genotypes [9]. The high sequence identity, with only a few amino acid substitutions between members of the cluster groups in our study and those reported previously throughout Mexico could be accounted for in several ways. Nucleotide changes at, for example, mutational hotspots, could evolve concurrently in different isolates from different regions. The probability that this explanation accounts for all observed genotypic changes in this study seems low. A more likely explanation is parasite gene flow across an endemic region through movement of infected cattle and vector migration on a mammalian host (e.g. wildlife). It is worth noting that a similar distribution of genotypes has also been found in a subset of *Plasmodium* infected individuals [11].

Among the five samples that contained multiple MSA-1 genotypes concurrently, one sample with multiple genotypes was detected at the first time point, while the remaining four were from the second sampling 6 months later. This finding is consistent with the previously reported oligoclonal structure of *B. bovis* populations in individual animals [15,21] and has also been reported in *Theileria* infected cattle within an endemic population [23]. Only a single MSA-1 genotype was detected in the respective first samplings of the four animals with multiple genotypes at second sampling. Although it is possible that genotypes present at the second sampling were initially missed due to a low infection level, re-testing of the DNA isolated from the same animal at the first sampling again failed to identify the second sampling genotypes in that animal. With a total of 60 clones sequenced from the first sample, our approach had a >95% probability of detecting genotypes present at ≥9.5% in the original sample. Thus, it is possible that genotypes “uniquely” present at the second sampling represent either genotypes present in the population at a level below 9.5%, or introduction of “new” genotypes in the population. While these new genotypes may have arisen within an individual animal through mutation, super-infection with a second unique genotype (resulting in co-infection) due to exposure to a tick vector population infected with one or more different MSA-1 genotypes seems a more likely explanation and is more consistent with a shift over time not only in genotype but also in cluster type. Examples of super-infection or co-infection have been reported in other parasites [3,7,8,17,20], and experimental transmission of multiple *B. bovis* strains with distinct MSA-1 genotypes by ticks has been demonstrated [4]. As noted above, we cannot rule out that the changes observed may reflect emergence of a genotype at a level below detection at the first sampling.

There were no reports of clinical disease in the study population during the sampling time period. This suggests that a buildup of MSA-1 sequence changes can result in expansion of a new genotype in the population without clinical disease. It is possible that a more extensive accumulation of minor mutational events, a major shift in MSA-1 through recombination in the tick vector, other changes in variant antigens such as variant erythrocyte surface antigens (VESA) [1], increased challenge pressure, or likely a combination of factors are necessary before introduction of a new strain can result in clinical disease within a host population that is persistently infected with and immune to existing strains. Evidence for determining which of these changes are important is currently lacking. However, the requirement for multiple changes and a major antigenic shift before selection of a breakthrough population occurs is consistent with the prolonged period required for the occurrence of vaccine breakthrough [15].

The extent of variation in the genotypes within this cohort was compared with vaccine breakthrough isolates from Australia previously analyzed and the locations of change within the hypervariable region are very similar, with conservation of sequence motifs, two of which were previously identified [14]. The overall percentage of amino acid identity between Australian and Mexican isolates ranges from 20% to 59%, while the percentage of amino acid identity within Australian isolates is broader, ranging from 12% to 99%, with evidence of recombination [3]. Currently, we cannot rule out if the MSA-1 sequence changes identified in this study were due to the recombination or a consequence of point mutations. Nevertheless, multiple and different strain variants may be two factors required for recombination within a parasite population, and the 28 unique MSA-1 sequence variants found within this population may provide a pool of templates that increase the probability of future recombination.

In conclusion, we report that the population diversity of *B. bovis* MSA-1 genotypes within a persistently infected bovine reservoir in an endemic region is broader than expected and fluctuates over time. No geographical clustering of *B. bovis* transmission was observed, a trait similarly found in other Apicomplexan parasites, and co-infection with multiple MSA-1 genotypes in several infected animals was detected. How and why such variability of MSA-1 sequences is generated in the reservoir or vector host is unknown. But it is likely that infection with one or more genotypically distinct strains circulating in the population can occur in persistently infected cattle, apparently without significant clinical disease. Co-infection with multiple strains within the mammalian host will increase the likelihood of transmission by co-infected ticks, and increase the pool of templates for recombination that occurs in sexual stages within the tick vector. Buildup of antigenic diversity and variation in this manner, not only of MSA-1, but also of other divergent proteins, may ultimately result in breakthrough of immunity in persistently infected animals, whether vaccinated or naturally infected.

## Figures and Tables

**Table 1:** MSA-1 genotype analysis of 20 of 31 animals that were tracked individually between samplings and had MSA-1 inter- or intra-group genotype shifts.

Animal ID	1st sampling (group^a^)	2nd sampling (group^a^)	Inter-group changes	Genotype (G) shifts
64	1	1	No	G5 to G1
1527	1	1	No	G3 to G1
1530	1	1	No	G6 to G14
1546	1	1	No	G4 to G9
1549	1	1	No	G1 to G3
2088	1	1	No	G7 to G1, 14, 23–26
3539	1	1	No	G8 to G1
3571	1	1	No	G2 to G3
3611	1	1	No	G2 to G1
3616	3	1	Yes	G20 to G14
4504	1	2	Yes	G2 to G18
4506	1	1	No	G2 to G1
4507	2	1	Yes	G15 to G1
4509	1	1	No	G3 to G2
4526	3	1	Yes	G19 to G1, 2
5503	1	1	No	G2 to G3
5545	1	1	No	G2 to G14
5585	2	1	Yes	G15 to G1, 2, 27, 28
5591	2	1	Yes	G17 to G2
8513	2	1	Yes	G15 to G12

a Group 1 genotypes include 1–14, 21–28; group 2 includes genotypes 15–18 and group 3 includes genotypes 19–20.

## Appendix A Supplementary data

Fig. S1. Multiple deduced amino acid sequence alignment of *B. bovis* MSA-1 of the 28 genotypes isolated within a single cattle cohort in Nayarit, Mexico. Grey shaded areas represent conserved sequence homology and red opaque regions show some conserved motifs with unknown function. Genotypes group clusters were shaded as follows: pink, green and blue are group clusters 1, 2 and 3, respectively.

Fig. S2. Multiple deduced amino acid sequence alignment of *B. bovis* MSA-1 between genotype 2 identified in our study and known MSA-1 isolated in Chiapas, Jalisco and Tabasco states in Mexico. Sequence differences appear to be randomly distributed.

## References

[1] Al-Khedery B., Allred D.R. (2006). Antigenic variation in Babesia bovis occurs through segmental gene conversion of the ves multigene family, within a bidirectional locus of active transcription. Mol Microbiol.

[2] Allred D.R., Al-Khedery B. (2004). Antigenic variation and cytoadhesion in Babesia bovis and Plasmodium falciparum: different logics achieve the same goal. Mol Biochem Parasitol.

[3] Bazarusanga T., Vercruysse J., Marcotty T., Geysen D. (2007). Epidemiological studies on Theileriosis and the dynamics of Theileria parva infections in Rwanda. Vet Parasitol.

[4] Berens S.J., Brayton K.A., McElwain T.F. (2007). Coinfection with antigenically and genetically distinct virulent strains of Babesia bovis is maintained through all phases of the parasite life cycle. Infect Immun.

[5] Berens S.J., Brayton K.A., Molloy J.B., Bock R.E., Lew A.E., McElwain T.F. (2005). Merozoite surface antigen 2 proteins of Babesia bovis vaccine breakthrough isolates contain a unique hypervariable region composed of degenerate repeats. Infect Immun.

[6] Brown W.C., Palmer G.H., McElwain T.F., Hines S.A., Dobbelaere D.A. (1993). Babesia bovis: characterization of the T helper cell response against the 42-kDa merozoite surface antigen (MSA-1) in cattle. Exp Parasitol.

[7] Cordon-Obras C., Berzosa P., Ndong-Mabale N. (2009). Trypanosoma brucei gambiense in domestic livestock of Kogo and Mbini foci (Equatorial Guinea). Trop Med Int Health.

[8] Futse J.E., Brayton K.A., Dark M.J., Knowles D.P., Palmer G.H. (2008). Superinfection as a driver of genomic diversification in antigenically variant pathogens. Proc Natl Acad Sci U S A.

[9] Genis A.D., Mosqueda J.J., Borgonio V.M. (2008). Phylogenetic analysis of Mexican Babesia bovis isolates using msa and ssrRNA gene sequences. Ann N Y Acad Sci.

[10] Hines S.A., Palmer G.H., Jasmer D.P., McGuire T.C., McElwain T.F. (1992). Neutralization-sensitive merozoite surface antigens of Babesia bovis encoded by members of a polymorphic gene family. Mol Biochem Parasitol.

[11] Joshi H., Valecha N., Verma A. (2007). Genetic structure of Plasmodium falciparum field isolates in eastern and north-eastern India. Malar J.

[12] Lau A.O. (2009). An overview of the Babesia, Plasmodium and Theileria genomes: a comparative perspective. Mol Biochem Parasitol.

[13] LeRoith T., Berens S.J., Brayton K.A. (2006). The Babesia bovis merozoite surface antigen 1 hypervariable region induces surface-reactive antibodies that block merozoite invasion. Infect Immun.

[14] Leroith T., Brayton K.A., Molloy J.B. (2005). Sequence variation and immunologic cross-reactivity among Babesia bovis merozoite surface antigen 1 proteins from vaccine strains and vaccine breakthrough isolates. Infect Immun.

[15] Lew A.E., Bock R.E., Croft J.M., Minchin C.M., Kingston T.G., Dalgliesh R.J. (1997). Genotypic diversity in field isolates of Babesia bovis from cattle with babesiosis after vaccination. Aust Vet J.

[16] Palmer G.H., McElwain T.F., Perryman L.E. (1991). Strain variation of Babesia bovis merozoite surface-exposed epitopes. Infect Immun.

[17] Peacock L., Ferris V., Bailey M., Gibson W. (2007). Dynamics of infection and competition between two strains of Trypanosoma brucei brucei in the tsetse fly observed using fluorescent markers. Kinetoplastid Biol Dis.

[18] Radke J.R., Behnke M.S., Mackey A.J., Radke J.B., Roos D.S., White M.W. (2005). The transcriptome of Toxoplasma gondii. BMC Biol.

[19] Shkap V., Pipano E., McElwain T.F. (1994). Cross-protective immunity induced by Babesia bovis clones with antigenically unrelated variable merozoite surface antigens. Vet Immunol Immunopathol.

[20] Smith T., Felger I., Beck H.P., Tanner M. (1999). Consequences of multiple infection with Plasmodium falciparum in an area of high endemicity. Parassitologia.

[21] Stich R.W., Rice-Ficht A.C., Tuo W., Brown W.C. (1999). Babesia bovis: common protein fractions recognized by oligoclonal B. bovis-specific CD4+ T cell lines from genetically diverse cattle. Exp Parasitol.

[22] Suarez C.E., McElwain T.F., Echaide I., Torioni de Echaide S., Palmer G.H. (1994). Interstrain conservation of babesial RAP-1 surface-exposed B-cell epitopes despite rap-1 genomic polymorphism. Infect Immun.

[23] Weir W., Ben-Miled L., Karagenc T. (2007). Genetic exchange and sub-structuring in Theileria annulata populations. Mol Biochem Parasitol.

[24] Zhuang W.Z., Sugimoto C., Kubota S., Onoe S., Onuma M. (1995). Antigenic alteration in major piroplasm surface proteins of Theileria sergenti during infection. Vet Parasitol.

