# Supplementary material for: Genotypic diversity of merozoite surface antigen 1 of Babesia bovis within an endemic population
Source: Mol Biochem Parasitol. 2010 Aug;172(2-2):107–12. doi: 10.1016/j.molbiopara.2010.03.017 (PMC2941823; doi:10.1016/j.molbiopara.2010.03.017)
Supplement: Supplementary file 1 — Fig. S1. Multiple deduced amino acid sequence alignment of B. bovis MSA-1 of the 28 genotypes isolated within a single cattle cohort in Nayarit, Mexico. Grey shaded areas represent conserved sequence homology and red opaque regions show some conserved motifs with unknown function. Genotypes group clusters were shaded as follows: pink, green and blue are group clusters 1, 2 and 3, respectively. [file mmc1.pdf]

Figure S1.

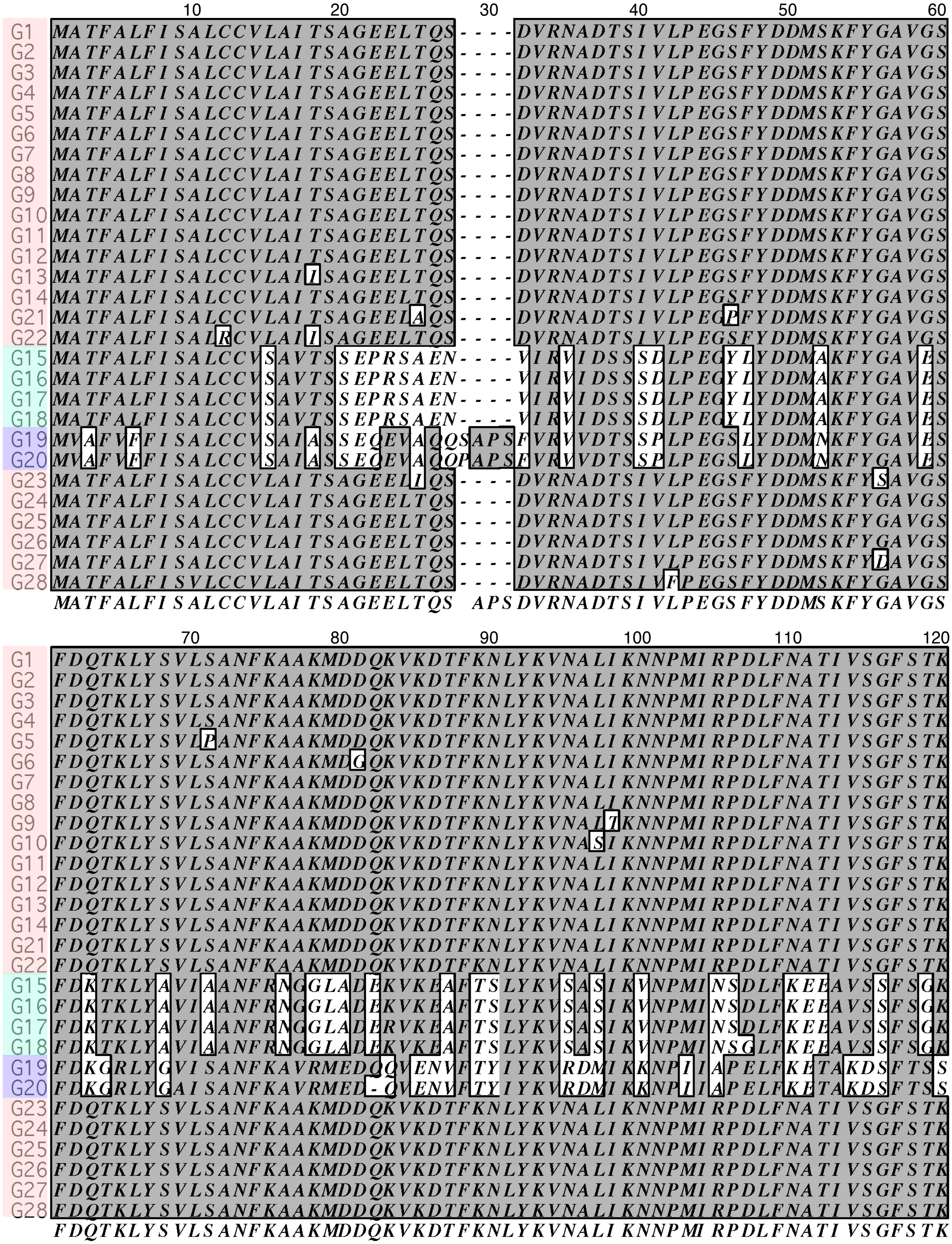
10 20 30 40 50 60
G1 MATFALFISALCCVLAITSAGEELTQS----DVRNADTSIVLPEGSFYDDMSKFYGAVGS
G2 MATFALFISALCCVLAITSAGEELTQS----DVRNADTSIVLPEGSFYDDMSKFYGAVGS
G3 MATFALFISALCCVLAITSAGEELTQS----DVRNADTSIVLPEGSFYDDMSKFYGAVGS
G4 MATFALFISALCCVLAITSAGEELTQS----DVRNADTSIVLPEGSFYDDMSKFYGAVGS
G5 MATFALFISALCCVLAITSAGEELTQS----DVRNADTSIVLPEGSFYDDMSKFYGAVGS
G6 MATFALFISALCCVLAITSAGEELTQS----DVRNADTSIVLPEGSFYDDMSKFYGAVGS
G7 MATFALFISALCCVLAITSAGEELTQS----DVRNADTSIVLPEGSFYDDMSKFYGAVGS
G8 MATFALFISALCCVLAITSAGEELTQS----DVRNADTSIVLPEGSFYDDMSKFYGAVGS
G9 MATFALFISALCCVLAITSAGEELTQS----DVRNADTSIVLPEGSFYDDMSKFYGAVGS
G10 MATFALFISALCCVLAITSAGEELTQS----DVRNADTSIVLPEGSFYDDMSKFYGAVGS
G11 MATFALFISALCCVLAITSAGEELTQS----DVRNADTSIVLPEGSFYDDMSKFYGAVGS
G12 MATFALFISALCCVLAITSAGEELTQS----DVRNADTSIVLPEGSFYDDMSKFYGAVGS
G13 MATFALFISALCCVLAIISAGEELTQS----DVRNADTSIVLPEGSFYDDMSKFYGAVGS
G14 MATFALFISALCCVLAITSAGEELTQS----DVRNADTSIVLPEGSFYDDMSKFYGAVGS
G21 MATFALFISALCCVLAITSAGEELAQS----DVRNADTSIVLPEGPFYDDMSKFYGAVGS
G22 MATFALFISALRCVLAIISAGEELTQS----DVRNADTSIVLPEGSFYDDMSKFYGAVGS
G15 MATFALFISALCCVSAVTSSEPRSAEN----VIRVIDSSSDLPEGYLYDDMAKFYGAVES
G16 MATFALFISALCCVSAVTSSEPRSAEN----VIRVIDSSSDLPEGYLYDDMAKFYGAVES
G17 MATFALFISALCCVSAVTSSEPRSAEN----VIRVIDSSSDLPEGYLYDDMAKFYGAVES
G18 MATFALFISALCCVSAVTSSEPRSAEN----VIRVIDSSSDLPEGYLYDDMAKFYGAVES
G19 MVAFVFFISALCCVSAIASSEQEVAQQSAPSFVRVVDTSSPLPEGSLYDDMNKFYGAVES
G20 MVAFVFFISALCCVSAIASSEQEVAQQPAPSFVRVVDTSSPLPEGSLYDDMNKFYGAVES
G23 MATFALFISALCCVLAITSAGEELIQS----DVRNADTSIVLPEGSFYDDMSKFYSAVGS
G24 MATFALFISALCCVLAITSAGEELTQS----DVRNADTSIVLPEGSFYDDMSKFYGAVGS
G25 MATFALFISALCCVLAITSAGEELTQS----DVRNADTSIVLPEGSFYDDMSKFYGAVGS
G26 MATFALFISALCCVLAITSAGEELTQS----DVRNADTSIVLPEGSFYDDMSKFYGAVGS
G27 MATFALFISALCCVLAITSAGEELTQS----DVRNADTSIVLPEGSFYDDMSKFYDAVGS
G28 MATFALFISVLCCVLAITSAGEELTQS----DVRNADTSIVFPEGSFYDDMSKFYGAVGS
MATFALFISALCCVLAITSAGEELTQS APSDVRNADTSIVLPEGSFYDDMSKFYGAVGS
70 80 90 100 110 120
G1 FDQTKLYSVLSANFKAAKMDDQKVKDTFKNLYKVNALIKNNPMIRPDLFNATIVSGFSTK
G2 FDQTKLYSVLSANFKAAKMDDQKVKDTFKNLYKVNALIKNNPMIRPDLFNATIVSGFSTK
G3 FDQTKLYSVLSANFKAAKMDDQKVKDTFKNLYKVNALIKNNPMIRPDLFNATIVSGFSTK
G4 FDQTKLYSVLSANFKAAKMDDQKVKDTFKNLYKVNALIKNNPMIRPDLFNATIVSGFSTK
G5 FDQTKLYSVLPANFKAAKMDDQKVKDTFKNLYKVNALIKNNPMIRPDLFNATIVSGFSTK
G6 FDQTKLYSVLSANFKAAKMDGQKVKDTFKNLYKVNALIKNNPMIRPDLFNATIVSGFSTK
G7 FDQTKLYSVLSANFKAAKMDDQKVKDTFKNLYKVNALIKNNPMIRPDLFNATIVSGFSTK
G8 FDQTKLYSVLSANFKAAKMDDQKVKDTFKNLYKVNALIKNNPMIRPDLFNATIVSGFSTK
G9 FDQTKLYSVLSANFKAAKMDDQKVKDTFKNLYKVNALTKNNPMIRPDLFNATIVSGFSTK
G10 FDQTKLYSVLSANFKAAKMDDQKVKDTFKNLYKVNASIKNNPMIRPDLFNATIVSGFSTK
G11 FDQTKLYSVLSANFKAAKMDDQKVKDTFKNLYKVNALIKNNPMIRPDLFNATIVSGFSTK
G12 FDQTKLYSVLSANFKAAKMDDQKVKDTFKNLYKVNALIKNNPMIRPDLFNATIVSGFSTK
G13 FDQTKLYSVLSANFKAAKMDDQKVKDTFKNLYKVNALIKNNPMIRPDLFNATIVSGFSTK
G14 FDQTKLYSVLSANFKAAKMDDQKVKDTFKNLYKVNALIKNNPMIRPDLFNATIVSGFSTK
G21 FDQTKLYSVLSANFKAAKMDDQKVKDTFKNLYKVNALIKNNPMIRPDLFNATIVSGFSTK
G22 FDQTKLYSVLSANFKAAKMDDQKVKDTFKNLYKVNALIKNNPMIRPDLFNATIVSGFSTK
G15 FDKTKLYAVIAANFRNGGLADEKVKEAFTSLYKVSASIKVNPMINSDLFKEEAVSSFSGK
G16 FDKTKLYAVIAANFRNGGLADEKVKEAFTSLYKVSASIKVNPMINSDLFKEEAVSSFSGK
G17 FDKTKLYAVIAANFRNGGLADERVKEAFTSLYKVSASIKVNPMINSDLFKEEAVSSFSGK
G18 FDKTKLYAVIAANFRNGGLADEKVKEAFTSLYKVSASIKVNPMINSGLFKEEAVSSFSGK
G19 FDKGRLYGVISANFKAVRMEDQQVENVFTYIYKVRDMIKKNPIIAPELFKETAKDSFTSS
G20 FDKGRLYGAISANFKAVRMED-QVENVFTYIYKVRDMIKKNPIIAPELFKETAKDSFTSS
G23 FDQTKLYSVLSANFKAAKMDDQKVKDTFKNLYKVNALIKNNPMIRPDLFNATIVSGFSTK
G24 FDQTKLYSVLSANFKAAKMDDQKVKDTFKNLYKVNALIKNNPMIRPDLFNATIVSGFSTK
G25 FDQTKLYSVLSANFKAAKMDDQKVKDTFKNLYKVNALIKNNPMIRPDLFNATIVSGFSTK
G26 FDQTKLYSVLSANFKAAKMDDQKVKDTFKNLYKVNALIKNNPMIRPDLFNATIVSGFSTK
G27 FDQTKLYSVLSANFKAAKMDDQKVKDTFKNLYKVNALIKNNPMIRPDLFNATIVSGFSTK
G28 FDQTKLYSVLSANFKAAKMDDQKVKDTFKNLYKVNALIKNNPMIRPDLFNATIVSGFSTK
FDQTKLYSVLSANFKAAKMDDQKVKDTFKNLYKVNALIKNNPMIRPDLFNATIVSGFSTK

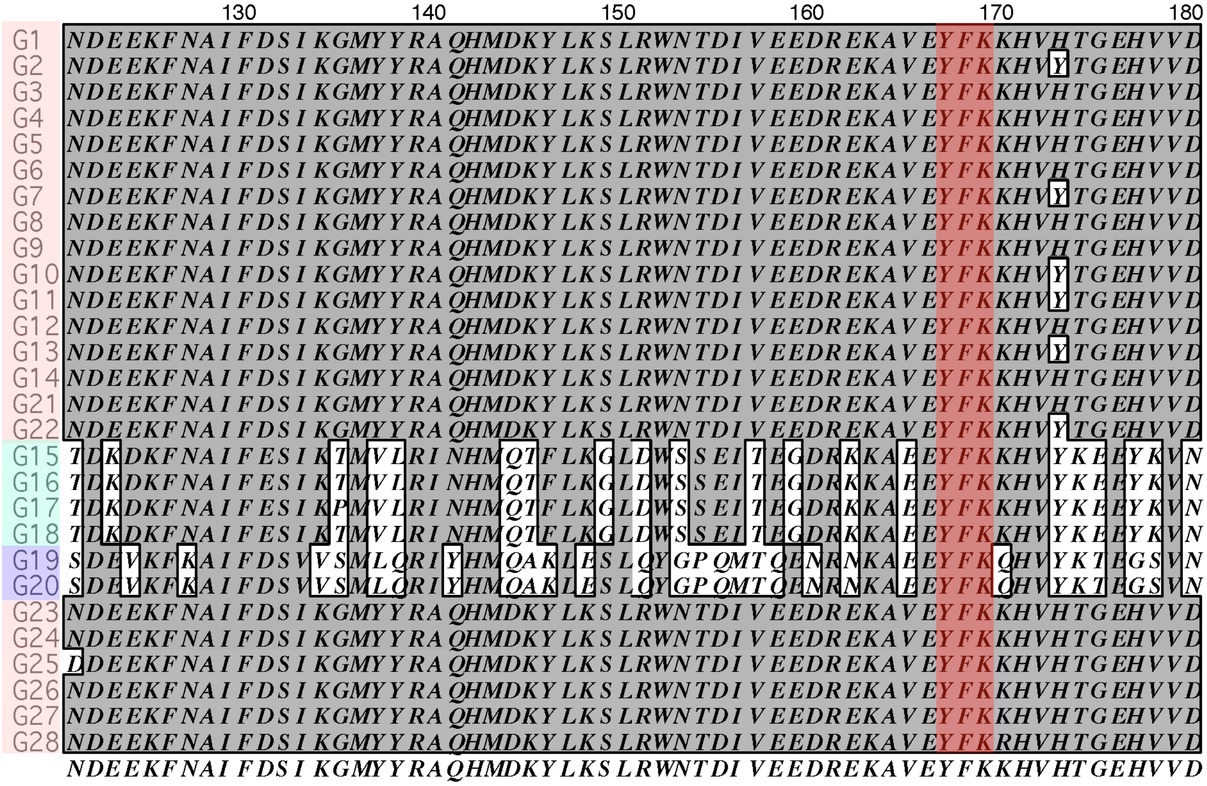
130 140 150 160 170 180
G1 NDEEKFNAIFDSIKGMYYRAQHMDKYLKSLRWNTDIVEEDREKAVEYFKKHVHTGEHVVD
G2 NDEEKFNAIFDSIKGMYYRAQHMDKYLKSLRWNTDIVEEDREKAVEYFKKHVYTGEHVVD
G3 NDEEKFNAIFDSIKGMYYRAQHMDKYLKSLRWNTDIVEEDREKAVEYFKKHVHTGEHVVD
G4 NDEEKFNAIFDSIKGMYYRAQHMDKYLKSLRWNTDIVEEDREKAVEYFKKHVHTGEHVVD
G5 NDEEKFNAIFDSIKGMYYRAQHMDKYLKSLRWNTDIVEEDREKAVEYFKKHVHTGEHVVD
G6 NDEEKFNAIFDSIKGMYYRAQHMDKYLKSLRWNTDIVEEDREKAVEYFKKHVHTGEHVVD
G7 NDEEKFNAIFDSIKGMYYRAQHMDKYLKSLRWNTDIVEEDREKAVEYFKKHVYTGEHVVD
G8 NDEEKFNAIFDSIKGMYYRAQHMDKYLKSLRWNTDIVEEDREKAVEYFKKHVHTGEHVVD
G9 NDEEKFNAIFDSIKGMYYRAQHMDKYLKSLRWNTDIVEEDREKAVEYFKKHVHTGEHVVD
G10 NDEEKFNAIFDSIKGMYYRAQHMDKYLKSLRWNTDIVEEDREKAVEYFKKHVYTGEHVVD
G11 NDEEKFNAIFDSIKGMYYRAQHMDKYLKSLRWNTDIVEEDREKAVEYFKKHVYTGEHVVD
G12 NDEEKFNAIFDSIKGMYYRAQHMDKYLKSLRWNTDIVEEDREKAVEYFKKHVHTGEHVVD
G13 NDEEKFNAIFDSIKGMYYRAQHMDKYLKSLRWNTDIVEEDREKAVEYFKKHVYTGEHVVD
G14 NDEEKFNAIFDSIKGMYYRAQHMDKYLKSLRWNTDIVEEDREKAVEYFKKHVHTGEHVVD
G21 NDEEKFNAIFDSIKGMYYRAQHMDKYLKSLRWNTDIVEEDREKAVEYFKKHVHTGEHVVD
G22 NDEEKFNAIFDSIKGMYYRAQHMDKYLKSLRWNTDIVEEDREKAVEYFKKHVYTGEHVVD
G15 TDKDKFNAIFESIKTMVLRINHMQTFLKGLDWSSEITEGDRKKAEEYFKKHVYKEEYKVN
G16 TDKDKFNAIFESIKTMVLRINHMQTFLKGLDWSSEITEGDRKKAEEYFKKHVYKEEYKVN
G17 TDKDKFNAIFESIKPMVLRINHMQTFLKGLDWSSEITEGDRKKAEEYFKKHVYKEEYKVN
G18 TDKDKFNAIFESIKTMVLRINHMQTFLKGLDWSSEITEGDRKKAEEYFKKHVYKEEYKVN
G19 SDEVKFKAIFDSVVSMLQRIYHMQAKLESLQYGPQMTQENRNKAEEYFKQHVYKTEGSVN
G20 SDEVKFKAIFDSVVSMLQRIYHMQAKLESLQYGPQMTQENRNKAEEYFKQHVYKTEGSVN
G23 NDEEKFNAIFDSIKGMYYRAQHMDKYLKSLRWNTDIVEEDREKAVEYFKKHVHTGEHVVD
G24 NDEEKFNAIFDSIKGMYYRAQHMDKYLKSLRWNTDIVEEDREKAVEYFKKHVHTGEHVVD
G25 DDEEKFNAIFDSIKGMYYRAQHMDKYLKSLRWNTDIVEEDREKAVEYFKKHVHTGEHVVD
G26 NDEEKFNAIFDSIKGMYYRAQHMDKYLKSLRWNTDIVEEDREKAVEYFKKHVHTGEHVVD
G27 NDEEKFNAIFDSIKGMYYRAQHMDKYLKSLRWNTDIVEEDREKAVEYFKKHVHTGEHVVD
G28 NDEEKFNAIFDSIKGMYYRAQHMDKYLKSLRWNTDIVEEDREKAVEYFKRHVHTGEHVVD
NDEEKFNAIFDSIKGMYYRAQHMDKYLKSLRWNTDIVEEDREKAVEYFKKHVHTGEHVVD

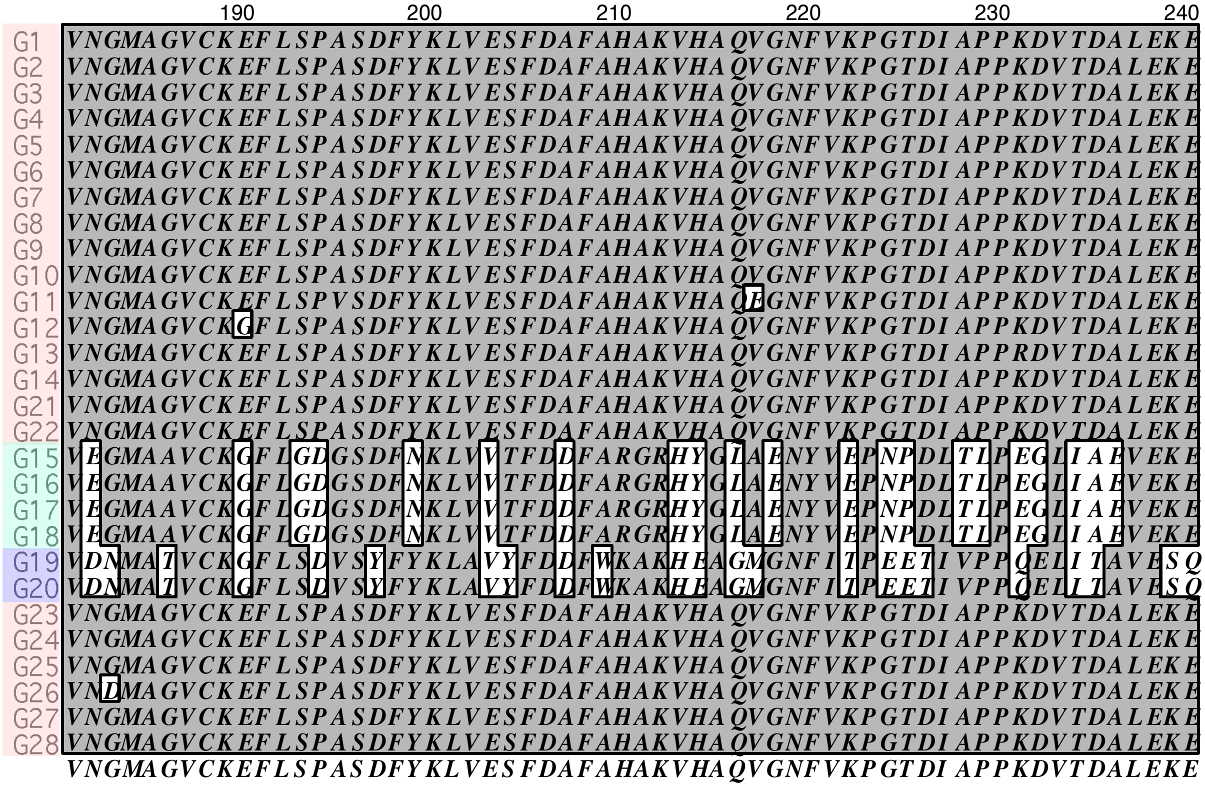
190 200 210 220 230 240
G1 VNGMAGVCKEFLSPASDFYKLVESFDAFAHAKVHAQVGNFVKPGTDIAPPKDVTDALEKE
G2 VNGMAGVCKEFLSPASDFYKLVESFDAFAHAKVHAQVGNFVKPGTDIAPPKDVTDALEKE
G3 VNGMAGVCKEFLSPASDFYKLVESFDAFAHAKVHAQVGNFVKPGTDIAPPKDVTDALEKE
G4 VNGMAGVCKEFLSPASDFYKLVESFDAFAHAKVHAQVGNFVKPGTDIAPPKDVTDALEKE
G5 VNGMAGVCKEFLSPASDFYKLVESFDAFAHAKVHAQVGNFVKPGTDIAPPKDVTDALEKE
G6 VNGMAGVCKEFLSPASDFYKLVESFDAFAHAKVHAQVGNFVKPGTDIAPPKDVTDALEKE
G7 VNGMAGVCKEFLSPASDFYKLVESFDAFAHAKVHAQVGNFVKPGTDIAPPKDVTDALEKE
G8 VNGMAGVCKEFLSPASDFYKLVESFDAFAHAKVHAQVGNFVKPGTDIAPPKDVTDALEKE
G9 VNGMAGVCKEFLSPASDFYKLVESFDAFAHAKVHAQVGNFVKPGTDIAPPKDVTDALEKE
G10 VNGMAGVCKEFLSPASDFYKLVESFDAFAHAKVHAQVGNFVKPGTDIAPPKDVTDALEKE
G11 VNGMAGVCKEFLSPVSDFYKLVESFDAFAHAKVHAQEGNFVKPGTDIAPPKDVTDALEKE
G12 VNGMAGVCKGFLSPASDFYKLVESFDAFAHAKVHAQVGNFVKPGTDIAPPKDVTDALEKE
G13 VNGMAGVCKEFLSPASDFYKLVESFDAFAHAKVHAQVGNFVKPGTDIAPPRDVTDALEKE
G14 VNGMAGVCKEFLSPASDFYKLVESFDAFAHAKVHAQVGNFVKPGTDIAPPKDVTDALEKE
G21 VNGMAGVCKEFLSPASDFYKLVESFDAFAHAKVHAQVGNFVKPGTDIAPPKDVTDALEKE
G22 VNGMAGVCKEFLSPASDFYKLVESFDAFAHAKVHAQVGNFVKPGTDIAPPKDVTDALEKE
G15 VEGMAAVCKGFLGDGSDFNKLVVTFDDFARGRHYGLAENYVEPNPDLTLPEGLIAEVEKE
G16 VEGMAAVCKGFLGDGSDFNKLVVTFDDFARGRHYGLAENYVEPNPDLTLPEGLIAEVEKE
G17 VEGMAAVCKGFLGDGSDFNKLVVTFDDFARGRHYGLAENYVEPNPDLTLPEGLIAEVEKE
G18 VEGMAAVCKGFLGDGSDFNKLVVTFDDFARGRHYGLAENYVEPNPDLTLPEGLIAEVEKE
G19 VDNMATVCKGFLSDVSYFYKLAVYFDDFWKAKHEAGMGNFITPEETIVPPQELITAVESQ
G20 VDNMATVCKGFLSDVSYFYKLAVYFDDFWKAKHEAGMGNFITPEETIVPPQELITAVESQ
G23 VNGMAGVCKEFLSPASDFYKLVESFDAFAHAKVHAQVGNFVKPGTDIAPPKDVTDALEKE
G24 VNGMAGVCKEFLSPASDFYKLVESFDAFAHAKVHAQVGNFVKPGTDIAPPKDVTDALEKE
G25 VNGMAGVCKEFLSPASDFYKLVESFDAFAHAKVHAQVGNFVKPGTDIAPPKDVTDALEKE
G26 VNDMAGVCKEFLSPASDFYKLVESFDAFAHAKVHAQVGNFVKPGTDIAPPKDVTDALEKE
G27 VNGMAGVCKEFLSPASDFYKLVESFDAFAHAKVHAQVGNFVKPGTDIAPPKDVTDALEKE
G28 VNGMAGVCKEFLSPASDFYKLVESFDAFAHAKVHAQVGNFVKPGTDIAPPKDVTDALEKE
VNGMAGVCKEFLSPASDFYKLVESFDAFAHAKVHAQVGNFVKPGTDIAPPKDVTDALEKE

```
                250                 260                 270                 280                 290             300
G1   LQEQKPARSESTEVPAPGDASG----------VQQPPASGTSPQGPAPTTP-SPSPESSG
G2   LQEQKPARSESTEVPAPGDASG----------VQQPPASGTSPQGPAPTTP-SPSPESSG
G3   LQEQKPARSESTEVPAPGDASG----------VQQPPASGTSPQGPAPTTP-SPSPESSG
G4   LQEQKPARSESTEVPAPGDASG----------VQQPPASGTSPQGPAPTTP-SPSPESSG
G5   LQEQKPARSESTEVPAPGDASG----------VQQPPASGTSPQGPAPTTP-SPSPESSG
G6   LQEQKPARSESTEVPAPGDASG----------VQQPPASGTSPQGPAPTTP-SPSPESSG
G7   LQEQKPARSESTEVPAPGDASG----------VQQPPASGTSPQGPAPTTP-SPSPESSG
G8   LQGQKPARSESTEVPAPGDASG----------VQQPPASGTSPQGPAPTTP-SPSPESSG
G9   LQEQKPARSESTEVPAPGDASG----------VQQPPASGTSPQGPAPTTP-SPSPESSG
G10  LQEQKPARSESTEVPAPGDASG----------VQQPPASGTSPQGPAPTTP-SPSPESSG
G11  LQEQKPARSESTEVPAPGDASG----------VQQPPASGTSPQGPAPTTP-SPSPESSG
G12  LQEQKPARSESTEVPAPSDASG----------VQQPPASGTSPQGPAPTTP-SPSPESSG
G13  LQEQKPVRSESTEVPAPGDASG----------VQQPPASGTSPQGPAPTTP-SPSPESSG
G14  LQEQKPARSESTEVPAPGDASG----------VQQPPASGTSPQGPAPTTP-SPSPESSG
G21  LQEQKPARSESTEVPAPGDASG----------VQQPPASGTSPQGPAPTTP-SPSPESSG
G22  LQEQKPARSESTEVPAPGDASG----------VQQPPASGTSPQGPAPTTP-SPSPESSG
G15  VATVKPVERENNRSPGTGAVPQPPVDGQAQQEISTPPSSPSSPESQSPQTQGAENPSPDS
G16  VATVKPVERENNRSPGTGAVPQPPVDGQAQQEIPTPSSSPSSPESQSPQTQGAENPSPDS
G17  VATVKPVERENNRSPGTGAVPQPPVDGQAQQEISTPPSSPSSPESQSPQTQGAENPSPDS
G18  VATVKPVERENNRSPGTGAVPQPPVDGQAQQEISTPPSSPSSPESQSPQTQGAENPSPDS
G19  EEPGRTENGDSRPQGEAGSGSGDGVSVVPQTPGKDQPPATSPTVQPAVPNP-SQADQPTK
G20  EEPGRTENGDSRPQGEAGSGSGDGVSVVPQTPGKDQPPATSPTVQPAVPNP-SQADQPSL
G23  LQEQKPARSESTEVPAPGDASG----------VQQPPASGTSPQGPAPTTP-SPSPESSG
G24  LQEQKPARSESTEVPAPGDASG----------VQQPPASGTSLQGPAPTTP-SPSPESSG
G25  LQEQKPARSESTEVPAPGDASG----------VQQPPASGTSPQGPAPTTP-SPSPESSG
G26  LQEQKPARSESTEVPAPGDASG----------VQQPPASGTSPQGPAPTTP-SPSPESSG
G27  LQEQKPARSESTEVPAPGDASG----------VQQPPASGTSPQGPAPTTP-SPSPESSG
G28  LQEQKPARSESTEVPAPGDASG----------VQQPPASGTSPQGPAPTTP-SPSPESSG
     LQEQKPARSESTEVPAPGDASGPPVDGQAQQEVQQPPASGTSPQGPAPTTPGSPSPESSG

                310                 320                 330
G1   NLQGQQGTTKPAGSSFTYGGLTVATLCYFVLSAF
G2   NLQGQQGTTKPAGSSFTYGGLTVATLCYFVLSAF
G3   NLQGQQGTTKPAGSSFTYGGLTVATLCYFVLSAL
G4   NLQGQQGTTKPAGSSFTYGGLTVATLCYFVLSAL
G5   NLQGQQGTTKPAGSSFTYGGLTVATLCYFVLSAL
G6   NLQGQQGTTKPAGSSFTYGGLTVATLCYFVLSAL
G7   NLQGQQGTTKPAGSSFTYGGLTVATLCYFVLSAL
G8   NLQGQQG-TKPAGSSFTYGGLTVATLCYFVLSAL
G9   NLQGQQGTTKPAGSSFTYGGLTVATLCYFVLSAL
G10  NLQGQQGTTKPAGSSFTYGGLTVATLCYFVLSAL
G11  NLQGQQGTTKPAGSSFTYGGLTVATLCYFVLSAF
G12  NLQGQQGTTKPAGSSFTYGGLTVATLCYFVLSAL
G13  NLQGQQGTTKPAGSSFTYGGSTVATLCYFVLSAL
G14  NLQGQQGTTKPAGSSFTYGGLTVATLGYFVLSAF
G21  NLQGQQGTTKPAGSSFTYGGLTVATLCYFVLSAF
G22  NLQGQQGTTKPAGSSFTYGGLTVATLCYFVLSAF
G15  SQGNLNGQREPAKSSFTYGGLTVATLCYFVLSAF
G16  SQGNLNGQREPAKSSFTYGGLTVATLCYFVLSAF
G17  SQGNLNGQREPAKSSFTYGGLTVATLCYFVLSAF
G18  SQGNLNGQREPAKSSFTYGGLTVATLCYFVLSAF
G19  PEGNLNGQQGSPKPSFTFGGLTVATLCYFVLSAF
G20  KVTSMDNRVHPSLPLSPLADLWLLSATSFSLHF-
G23  NLQGQQGTTKPAGSSFTYGGLTVATLCYFVLSAF
G24  NLQGQQGTTKPAGSSFTYGGLTVATLCYFVLSAF
G25  NLQGQQGTTKPAGSSFTYGGLTVATLCYFVLSAF
G26  NLQGQQGTTKPAGSSFTYGGLTVATLCYFVLSAF
G27  NLQGQQGTTKPAGSSFTYGGLTVATLCYFVLSAF
G28  NLQGQQGTTKPAGSSFTYGGLTVATLCYFVLSAF
     NLQGQQGTTKPAGSSFTYGGLTVATLCYFVLSAF
```
