# Supplementary material for: Genotypic diversity of merozoite surface antigen 1 of Babesia bovis within an endemic population
Source: Mol Biochem Parasitol. 2010 Aug;172(2-2):107–12. doi: 10.1016/j.molbiopara.2010.03.017 (PMC2941823; doi:10.1016/j.molbiopara.2010.03.017)
Supplement: Supplementary file 2 — Fig. S2. Multiple deduced amino acid sequence alignment of B. bovis MSA-1 between genotype 2 identified in our study and known MSA-1 isolated in Chiapas, Jalisco and Tabasco states in Mexico. Sequence differences appear to be randomly distributed. [file mmc2.pdf]

Figure S2.

	1–30	31–60
Chiapas2	MATFALFISALCCVLAITSAGEELTQSDVR	NADTSIVLPEGSFYDDMSKFYGAVGSFDQT
Jalisco	MATFALFISALCCVLAITSAGEELTQSDVR	NADTSIV**P**PEGSFYDDMSKFYGAVGSFDQT
Tabasco	MATFALFISALCCVLAITSAGEELTQSDVR	NADTSIVLPEGSFYDDMSKFYGAVGSFDQT
Genotype 2	MATFALFISALCCVLAITSAGEELTQSDVR	NADTSIVLPEGSFYDDMSKFYGAVGSFDQT
	MATFALFISALCCVLAITSAGEELTQSDVR	NADTSIVLPEGSFYDDMSKFYGAVGSFDQT

	61–90	91–120
Chiapas2	KLYSVLSANFKAAKMD**G**QKVKDTFKNLYKV	NALIKNNPMIRPDLFNATIVSGFSTKNDEE
Jalisco	KLYSVLSANFKAAKMDDQKVKDTFKNLYKV	NALIKNNPMIRPDLFNATIVSGFSTKNDEE
Tabasco	KLYSVLSANFKAAKMDDQKVKDTFKNLYKV	NALIKNNPMIRPDLFNATIVSGFSTKNDEE
Genotype 2	KLYSVLSANFKAAKMDDQKVKDTFKNLYKV	NALIKNNPMIRPDLFNATIVSGFSTKNDEE
	KLYSVLSANFKAAKMDDQKVKDTFKNLYKV	NALIKNNPMIRPDLFNATIVSGFSTKNDEE

	121–150	151–180
Chiapas2	KFNAIFDSIKGMYYRAQHMDKYLKSLRWNT	DIVEEDREKAVEYFKKHVYTGEHVVDVNGM
Jalisco	KFNAIFDSIKGMYYRAQHMDKYLKSLRWNT	DIVEEDREKAVEYFKKHVYTGEHVVDVNGM
Tabasco	KFNAIFDSIKGMYYRAQHMDKYLKSLRWNT	DIVEEDREKAVEYFKKHVYTGEHVVDVNGM
Genotype 2	KFNAIFDSIKGMYYRAQHMDKYLKSLRWNT	DIVEEDREKAVEYFKKHVYTGEHVVDVNGM
	KFNAIFDSIKGMYYRAQHMDKYLKSLRWNT	DIVEEDREKAVEYFKKHVYTGEHVVDVNGM

	181–210	211–240
Chiapas2	AGVCKEFLSPASDFYKLVESFDAFAHAKVH	AQVGNFVKPGTDIAPPKDVTDALEKELQEQ
Jalisco	AGVCKEFLSPASDFYKLVESFDAFAHAKVH	AQVGNFVKPGTDIAPPKDVTDALEKELQEQ
Tabasco	AGVCKEFLSPASDFYKLVESFDAFAHAKVH	AQVGNFVKPGTDIAPPKDVTDALEKELQEQ
Genotype 2	AGVCKEFLSPASDFYKLVESFDAFAHAKVH	AQVGNFVKPGTDIAPPKDVTDALEKELQEQ
	AGVCKEFLSPASDFYKLVESFDAFAHAKVH	AQVGNFVKPGTDIAPPKDVTDALEKELQEQ

	241–270	271–300
Chiapas2	KPARSESTEVPAPGDASGVQQPPASGTSPQ	GPAPTTPSPSPESSGNLQGQQGTTKPAGSS
Jalisco	KPARSESTEVPAPGDASGVQQPPASGTSPQ	GPAPTTPSPSPESSGNLQGQQGTTKPAGSS
Tabasco	KPARSEST**G**VPAPGDASGVQQPPASGTSPQ	GPAPTTPSPSPESSGNLQGQQGTTKPAGSS
Genotype 2	KPARSESTEVPAPGDASGVQQPPASGTSPQ	GPAPTTPSPSPESSGNLQGQQGTTKPAGSS
	KPARSESTEVPAPGDASGVQQPPASGTSPQ	GPAPTTPSPSPESSGNLQGQQGTTKPAGSS

	301–319
Chiapas2	FTYGGLTVATLCYFVLSAF
Jalisco	FTYGGLTVATLCYFVLSAF
Tabasco	FTYGGLTVATLCYFVLSAF
Genotype 2	FTYGGLTVATLCYFVLSAF
	FTYGGLTVATLCYFVLSAF
